# Caffeinated Energy Drink Formulations Differentially Impact Hydration Versus Water: Does Habitual Caffeine Intake or Biological Sex Matter?

**DOI:** 10.3390/nu17182913

**Published:** 2025-09-09

**Authors:** Melinda Millard-Stafford, Brian Hack, Alec Harp, Ella Smith

**Affiliations:** Exercise Physiology Laboratory, School of Biological Sciences, Georgia Institute of Technology, Atlanta, GA 30332, USA; bhack@gatech.edu (B.H.); ellasmith8068@gmail.com (E.S.)

**Keywords:** sodium, potassium, fluid balance, ORS, osmolality, diuresis

## Abstract

**Background/Objectives**: The beverage hydration index (BHI) assesses the relative hydration properties of beverages. Commercially available caffeinated energy drinks (with and without electrolytes or carbohydrates) were compared to water. **Methods:** Fourteen men and fourteen women completed four trials: 500 mL water plus either 500 mL water or caffeinated energy drink beverage (CAF) with 280 mg, CAF (280 mg) plus electrolytes (CAF + E), or CAF (106 mg) + E and carbohydrates (CAF + CE). Participants’ habitual caffeine intake (0–535 mg/day) was used to stratify users into naïve (<25 mg/day, *n* = 19) or regular users. **Results:** BHIs at 240 min for CAF (0.86 ± 0.16) and CAF + E (0.91 ± 0.16) were lower (*p* < 0.001) versus water (1.0 ± 0.0) and CAF + CE (1.01 ± 0.12). Urine mass with CAF and CAF + E was significantly greater (*p* < 0.01) by 244 g and 162 g versus CAF + CE. The % fluid retained at 240 min was lower (*p* < 0.001) for CAF (−71.2 ± 21.5%) and CAF + E (−65.1 ± 26.3%) versus CAF + CE (−46.4 ± 18.8%). Habitual intake status had no effect on the BHI (*p* = 0.827) between the naïve (0.92 ± 0.1) and habitual user group (0.93 ± 0.1) averaged across all three caffeinated beverages for 120 and 240 min. At 240 min, the drink (*p* < 0.001) and drink x sex interaction (*p* = 0.042) indicated women had higher BHI than men (*p* = 0.03) for caffeinated drinks despite higher relative fluid and caffeine dosages. **Conclusions:** A low-carbohydrate–electrolyte beverage with moderate caffeine had similar hydration properties compared to water; however, this differs from beverages with higher caffeine containing limited carbohydrate and/or electrolytes, which were inferior to water. Habitual caffeine intake had no apparent influence, but men and women exhibited differences in the diuretic response to energy drink consumption.

## 1. Introduction

Water is essential for life, which emphasizes the need for optimal hydration to maintain fluid balance [1]. As such, most of the daily total water requirements are derived from ingested beverages [2]. While drinking water is the most obvious contribution, other beverages also contribute to total fluid intake, particularly across the age span of the general population and especially for individuals with significant body water turnover. Salt, when added to a beverage, increases fluid retention, particularly after significant fluid and electrolyte loss via sweating [3,4,5]. Other macronutrients (e.g., carbohydrates) may be included to simultaneously restore both fluid balance and re-synthesize muscle glycogen in athletes [6,7,8].

Caffeinated beverages (e.g., tea, coffee) remain a popular contribution to the daily total fluid intake [9]. The beverage hydration index (BHI) assesses fluid retention as a model to compare the hydration potential of beverages relative to water [10]. BHI is a valid model to assess beverage hydration characteristics under well-controlled conditions when individuals are euhydrated (differing from rehydration protocols following exercise). Water as the control beverage is equivalent to a BHI ratio of 1.0; therefore, values lower than 1.0 would indicate less hydration properties compared to water. In the first BHI experiment, caffeinated beverages such as tea, coffee, and diet soda had a BHI less than 1.0 but were not statistically less hydrating than water [10]. Later, Maughan et al. [11] reported that 0 to 400 mg of caffeinated coffee did not elicit greater diuresis (or lower BHI) versus water.

Yet, it is commonly reported that caffeine acts as a diuretic, with increased diuresis leading to negative fluid balance compared to plain water [12]. This point has been somewhat contentious over the years. Caffeine may increase the glomerular filtration rate (mediated by adenosine receptors in the kidney) and also induce natriuresis by inhibiting Na^+^ reabsorption in the renal proximal tubules [13]. Acute caffeine ingestion (≥250 mg, ~2–3 cups of coffee) increases short-term urine output for those who are not regularly using caffeine, but the diuretic effect appears to be attenuated for individuals who regularly consume caffeinated beverages [12]. Another review [14] corroborated these conclusions that tolerance to caffeine reduces the likelihood of impaired fluid retention elicited by caffeinated beverages. A subsequent meta-analysis [15] indicated a small but significant effect size for caffeine’s diuretic action (equal to ~16% higher vs. non-caffeinated beverages), and this effect was observed to be greater at rest compared to during exercise. Under sedentary conditions, moderate caffeine intake may not substantially impair hydration in habitual users, although high doses can elevate fluid loss [11]. In addition, a greater effect for diuresis was observed [15] in females (ES = 0.75) compared to males (ES = 0.13). However, the impact of biological sex reportedly does not impact the reproducibility of establishing the BHI metric for beverages [16].

Therefore, our purpose was to examine the impact of commercially available caffeinated energy drink formulae (with and without carbohydrate and/or electrolytes) on hydration properties via the beverage hydration index (BHI). We hypothesized that caffeinated energy drink consumption (<300 mg) would not adversely influence fluid retention over 4 h in habitual users but would deviate (with impaired fluid retention) in caffeine-naïve individuals under these sedentary conditions. Furthermore, since the BHI uses absolute volumes of ingested beverages (1 L), we hypothesized a greater diuresis with caffeine in smaller-sized females with lower total body water compared to male participants, consistent with the previous meta-analysis [15].

## 2. Materials and Methods

### 2.1. Study Population

Twenty-eight adult men and women from the local college campus population completed the study after providing written informed consent. The study was approved by the Georgia Tech Institutional Review Board (Protocol Number: H23495, December 2023) adhering to guidelines from the Declaration of Helsinki. Participants were recruited from the college population with a range of physical activity levels (sedentary up through highly trained college athletes). The physical characteristics of participants are presented in Table 1. The experimental sessions took place from spring through summer months (April to August). Participants were excluded based upon obesity (>35% body fat for women, >30% for men), active infection (e.g., urinary tract), metabolic (e.g., diabetes), renal, endocrine (hypo-, hyper-thyroid) or digestive diseases (e.g., hepatitis, cirrhosis, irritable bowel), smoking history, and medications altering fluid balance (e.g., diuretics) or over-the-counter drugs (e.g., anti-histamines, stimulants). Dietary supplement use during the study was prohibited (except for a multi-vitamin-mineral supplement). Participants were not allowed to be on an active weight loss program during the study. Women did not participate if pregnant or trying to conceive but were not excluded if taking oral contraceptives or if they had abnormal menstrual cycles. The phase of the menstrual cycle was also not controlled for during their scheduled participation.

Habitual caffeine intake was not an inclusion criterion (i.e., both naïve and habitual users were recruited). Caffeine was not allowed on the day of the test protocol, along with alcohol-containing beverages. The designation for caffeine naïve (<25 mg/day) [17] was based on a 7-day caffeinated food/beverage frequency questionnaire [18,19] and converted to caffeine values based on published sources of caffeine [20,21]. Based on this cut point, the naïve group consisted of 19 participants, and habitual users comprised only 9 participants. Estimated caffeine intake ranged from 0 to 5.0 mg per kg body mass per day. The average caffeine intake for those participants not classified as “naïve” was 156.7 ± 122 mg/day, and only one participant exceeded a daily caffeine level (400 mg/d) considered to be high or unsafe [22].

Height, weight, and body composition via skinfold thickness for men and women [23,24] were obtained prior to the first experimental trial. Multi-frequency bioimpedance (InBody 770, Seoul, Republic of Korea) was used for total body water, along with an estimation [25] based on fat-free mass obtained with skinfolds. Table 1 indicates the mean (±SD) physical characteristics of participants completing all four trials.

### 2.2. Study Design

Our aim was to compare hydration properties for three popular commercially available energy drink beverages based on the collection of cumulative urine mass following one liter ingestion of the drinks. Two energy drinks were matched for caffeine level (typical of an average daily amount), but differing in electrolyte content (no vs. low sodium) while the third energy drink had approximately 1/3 of the caffeine content and also contained levels of electrolytes and sugar that are representative of oral rehydration solutions (ORS) by the World Health Organization [26].

This study was a single-blind, crossover, quasi-experimental design using a within-subject design (i.e., each participant completed all beverage trials). Since beverages were received in commercially available containers, one member of the investigative team during data collection was not blinded to the beverages, although a numeric code for each beverage was used while processing the data analysis. Participants were uninformed which beverage was being given to them but could identify the water control. A Latin Square design was used to allocate the four conditions in a counterbalanced order, determined a priori by the study coordinator with participants assigned following enrollment. Water and the 3 test drinks were nearly equally distributed (based on total participants completing all trials) to receive an equal number of times in the 1st through 4th order position.

All testing was conducted in thermoneutral conditions (21 °C) in the laboratory based at Georgia Tech. All participants arrived in the morning (0700–0900 h) with the time of day held constant. Test sessions were completed on non-consecutive days within about a six-week period. Participants were asked to avoid strenuous activity for 24 h before each test day and record 24 h dietary intake (including beverages). Participants were instructed to replicate this diet in their remaining visits. Instructions for the day before testing also included limiting higher-sodium foods and avoiding alcohol and caffeine.

### 2.3. Test Beverages

Four beverage treatment trials were completed by each participant. Tap water served as the control. The other three test beverages were commercially available energy drinks with different combinations of electrolytes and carbohydrates. The test beverages did not have identical coloring but were matched as closely as possible for flavor (mango). One caffeine energy (CAF) beverage was carbonated, Celsius Live Fit^®^ mango passionfruit (Celsius Holdings, Inc., Boca Raton, FL, USA). The caffeine with electrolytes (CAF + E) beverage was Prime Energy^®^ orange mango (PRIME Hydration LLC, Congo Brands, Louisville, KY, USA). It should be noted (as shown in Table 2) that CAF + E also contained some carbohydrates, about one-third of that found in CAF + CE. The caffeine with electrolytes and carbohydrates (CAF + CE) beverage was Liquid IV Energy Multiplier mango tamarind (Liquid IV, El Segundo, CA, USA, a Unilever Company) powder added to 500 ML water. The sodium in CAF + CE is approximately equivalent to 43.5 mmol and marketed to be similar to an ORS [27].

Beverage composition for key electrolytes (sodium and potassium) and osmolality was measured and verified in our lab (presented in Table 2). Osmolality was measured by freezing-point depression with a Micro-Osmette osmometer (Precision Systems, Inc., Natick, MA, USA) using the median of three values from 50 µL samples. Horiba LAQUA twin (Horiba, Kyoto, Japan) compact ion meters were used for sodium and potassium. Since these were commercially available beverages, there were small differences in vitamins and other substances, which may or may not have exerted unknown metabolic effects (in low amounts). The other nutrients listed on the beverage label included CAF—Ca^+^ 140 mg/L, Vitamin C 168 mg/L, Riboflavin 5 mg/L, Vitamin B6 168 mg/L, Vitamin B12 17 mcg/L, Niacin 56 mg/L, Riboflavin 5 mg/L, Biotin 84 mg/L, pantothenic acid 28 mg/L, chromium 140 mcg/L; CAF + E—Ca^+^ and Mg^+^ 157 mg/L, Vit B6 7 mg/L, Vitamin B12 16 mcg/L; and CAF + CE—Vitamin C 153 mg/L, Niacin 48 mg/L, Vitamin B6 5 mg/L, Vitamin B12 14 mcg/L, pantothenic acid 24 mg/L. In addition, CAF lists unspecified amounts of taurine and glucuronolactone, and CAF + E lists taurine, L-theanine, and glucuronolactone. Beverages were stored in a refrigerator for the duration of the study at 5 °C. All beverages were served from a sealed container at a temperature between 10 and 12 °C during the 30-minute drinking period.

### 2.4. Test Protocol

**Figure 1:**
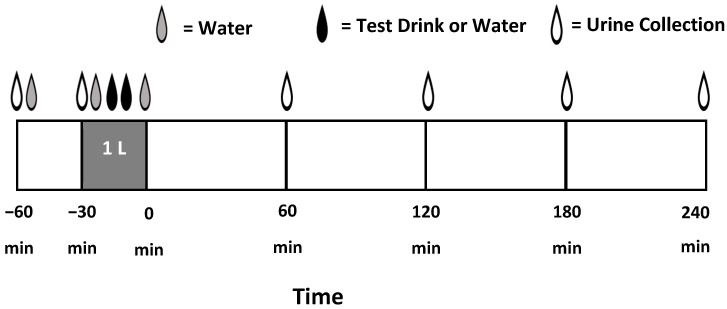
Schematic of the beverage hydration index test protocol performed for each beverage trial (*N* = 4 drink trials, 28 participants completing all trials).

The BHI protocol developed by Maughan et al. [10] was used to assess the relative hydration properties of the test beverages compared to water. A schematic of the BHI test protocol is depicted in Figure 1. The only modification from the original protocol was that instead of ingesting 1 L of each test energy drink, 500 mL of the test drink was ingested with the remaining 500 mL consumed as water. Participants’ pre-test dietary intake was not different across the four test sessions (as shown in Table 3). To ensure participants initiated each experimental trial in a state of euhydration, participants were requested to consume an additional 500 mL of water between 1800 and 2300 h in addition to their normal daily intake and then fast (food and fluid) until morning. Participants were also given 500 mL of tap water to consume 60 min prior to beginning each laboratory session (and the timing was repeated prior to each trial). A first-catch urine specimen was obtained in a sterile collection cup and transported to the lab at the start of each test session. Urine specific gravity (USG) was measured by refractometry (Atago, URC-Ne Refractometer, Tokyo, Japan), and mean USG values indicated euhydration across all trials (<1.020).

As depicted in Figure 1, participants were given one liter consisting of 250 mL water, the middle 500 mL of a test beverage (W, CAF, CAF + E, or CAF + CE), and the last 250 mL of water over 30 min (four times 250 mL aliquots every 7.5 min). After ingesting the beverage, participants remained seated, except for measuring body mass and urine output for 240 min after drinking the one-liter bolus. Body mass was obtained by having participants wear minimal clothing. Measurements were obtained hourly at 0, 60, 120, 180, and 240 min post-ingestion similar to other BHI protocols [10,16,28,29]. No food was consumed during the entire test session. Note that due to the use of an absolute volume of beverage (1000 mL) in the BHI protocol, the relative dosage of fluid was higher in women (16.3 mL/kg) compared to men (13.3 mL/kg). For the 280 mg caffeine beverage trials (CAF and CAF + E), the caffeine dose was 3.7 ± 0. 4 mg/kg for men and 4.8 ± 0.8 mg/kg for women.

Body mass and urine mass were measured on a digital platform scale to the gram (Ohaus Defender 5000, Model TD52P, Parsippany, NJ, USA). Male participants collected urine directly into a 3 L plastic 24 h urine container (Parter Medical Products, Carson, CA, USA), while the women utilized Vakly Graduated Specimen Collector Pans (Avaline Medical LLC, Lakewood, NJ, USA) placed under the toilet seat. Urine cumulative mass at each time point was measured on an electronic balance (to the nearest 0.1 g), with the mass of the empty container tared to subtract from total urine mass. BHI was calculated as the ratio of cumulative urine mass for tap water divided by the cumulative urine mass of the test beverage at each time point, as originally described [10]. Based on a previous paper [16], the clinically meaningful difference in BHI-derived hydration benefit is considered to be a ratio of 1.13 or 13% above water. Net fluid balance was calculated based on individual changes in body mass relative to the drink volume ingested. The % of the consumed fluid retained was calculated based on changes in body mass over time.

### 2.5. Statistical Analysis

A two-factor analysis of variance (ANOVA) with repeated measures (beverage × time) was used for all measures except the BHI. Since water is designated as the control (having a constant BHI score of 1.0 with no SD among subjects), the BHI comparison across all beverages required a non-parametric rank-based Friedman’s test [30] instead of ANOVA. To determine if the BHI of each energy test drink was inferior to the absolute water value of 1.0, a one-sample *t*-test was also performed. Data were analyzed using SPSS version 29 (IBM SPSS Statistics for Windows, Version 2.0. Armonk, NY, USA: IBM Corp). Post hoc comparisons were corrected using the Bonferroni method. An alpha level of *p* < 0.05 indicated statistical significance. Data are presented as mean (±SD) unless indicated otherwise.

Effect size calculations were based on previously published data [15], indicating a minimum sample size of *n* = 15 for four drink conditions. An additional sample size weighting was factored in to account for possible increased variance due to combining men and women. The final sample size estimate based on 80% power with a mean total urine output of 900 mL, a pooled SD of 300 mL, and a mean difference of 220 mL, detectable at an alpha level of 0.05, required a total of *n* = 20 observations per drink. Previous studies [18,19] have indicated no difference in BHI based on either sex or body mass. Therefore, fourteen men and fourteen women participated and were also analyzed by sub-group.

## 3. Results

### 3.1. Beverage Hydration Index (BHI)

**Figure 2:**
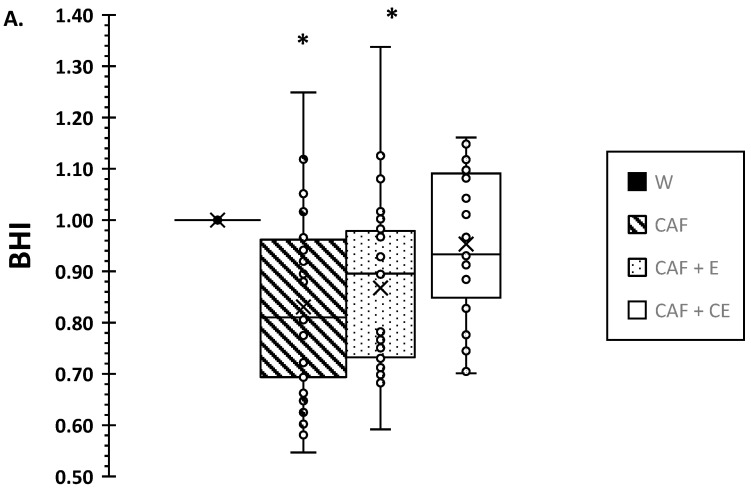

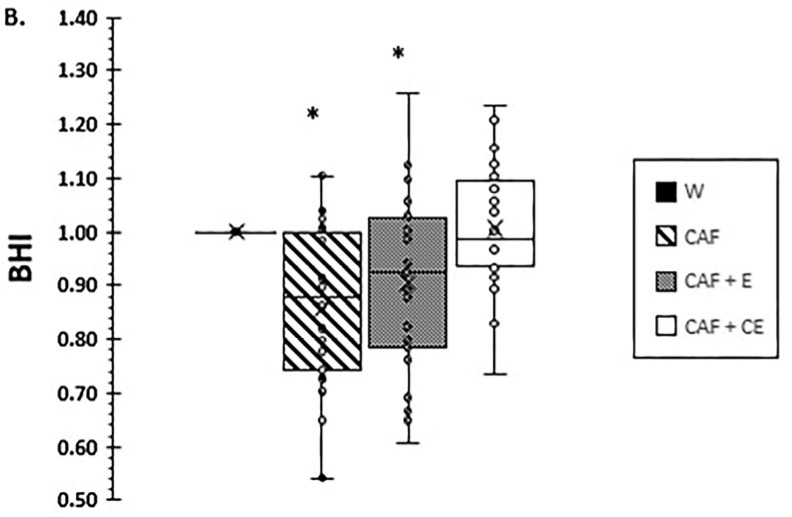
Box-and-whisker plot with individual values for beverage hydration index (BHI) at 120 min (**A**) and 240 min (**B**). Note that the BHI for water is a 1.0 ratio standard. X = mean values for beverages; horizontal line is the median. * lower BHI for energy drinks with higher caffeine (CAF) and caffeine + low levels of electrolytes (CAF + E) vs. water (W) and lower caffeine energy drink with carbohydrate and electrolytes (CAF + CE) (*p* < 0.001).

Figure 2 illustrates a box-and-whisker plot with mean, median, and individual BHI values for the four beverages at 120 and 240 min. BHI was significantly different among drinks (*p* < 0.001) with a drink × time interaction (*p* = 0.003). Both the CAF and CAF + E beverages had lower BHI vs. water (W) and CAF + CE (*p* = 0.001, *p* = 0.007). Water and CAF + CE did not differ at 120 min (*p* = 0.53) or 240 min (*p* = 1.0), although the BHI mean and median values for CAF + CE was <1.0 at 120 min and not significantly lower than 1.0 based on a one-sample *t* test (*p* = 0.089, 95% CI [−0.10, 0.007]). The BHI at 240 min for CAF was considered inferior (*p* < 0.001) compared to 1.0 for the water standard (mean difference of −13.6 and 95% CI lower bounds of the difference [−0.196, −0.075], which fell below the clinically relevant reference [16] of −0.13 (or −13%). The magnitude of this difference was large (Cohen’s d = −0.85). Similar results were also observed at 120 min for the BHI of CAF, which was inferior to 1.0 (mean difference of −16.5 and 95% CI lower bounds [−0.247, −0.08], d = −0.95). The BHIs at both 120 and 240 min for CAF + E were considered inferior (*p* < 0.001; *p* = 0.004) compared to 1.0 for the water standard (mean differences of −13.2 and −9.4) and 95% CI lower bounds [−0.21, −0.05] [−0.172, −0.017], with d = −0.79 and d = −0.595, respectively. Therefore, although CAF + CE provided no beneficial or detrimental effect on BHI compared to water, this was in contrast to the results for CAF and CAF + E.

**Figure 3:**
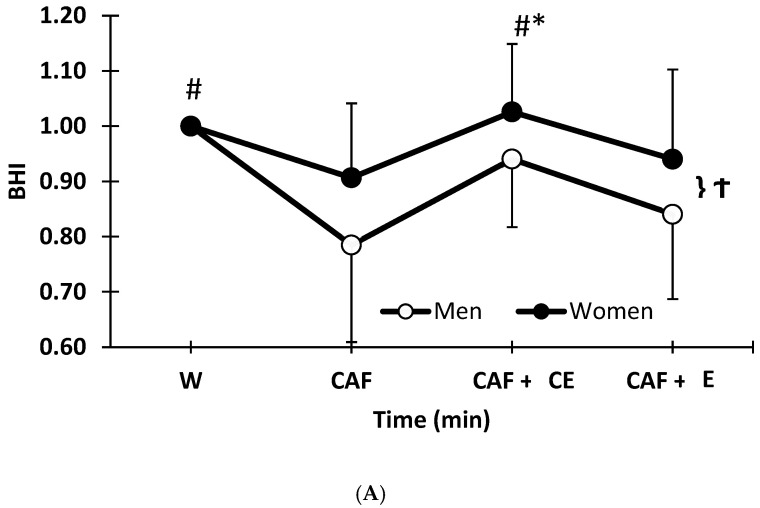

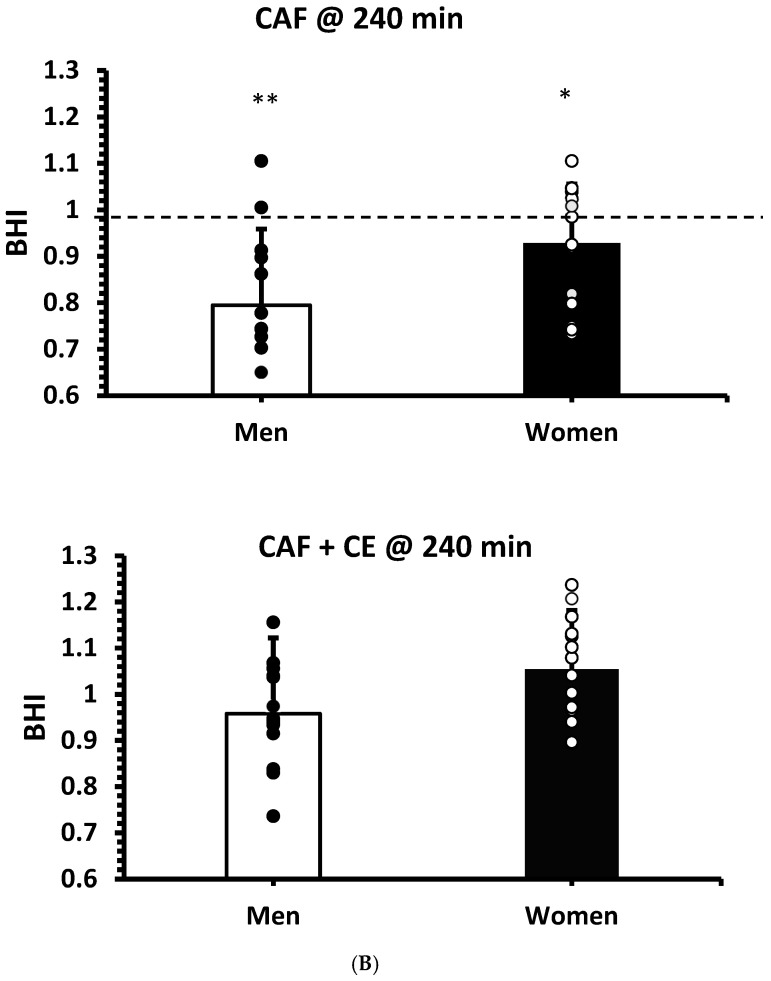
(**A**) Mean (SD) BHI differs based on sex at 240 min. † Women > Men within a caffeinated drink (*p* < 0.05). # For men, W and CAF + CE > CAF and CAF + E (*p* < 0.05). * For women, CAF + CE > CAF (*p* < 0.05). (**B**) Box plot with individual values for men and women in beverage hydration index (BHI) values for a higher-level caffeine energy drink (CAF), higher caffeine and low levels of electrolytes (CAF + E), and lower caffeine with electrolytes and carbohydrates (CAF + CE) compared to the water standard of 1.0 at 240 min. ** *p* < 0.001 compared to water control of 1.0; * *p* < 0.05 compared to water control of 1.0.

Sex effect on BHI. Women had a higher (*p* = 0.043) overall average BHI (across all beverages) compared to men by 0.077 (or 7.7% higher ratio than men) (Figure 3A). This sex effect was attributed to mean differences observed in CAF (*p* = 0.045) but not CAF + E (*p* = 0.093) or CAF + CE (*p* = 0.093). For men, both water and CAF + CE had overall greater BHI compared to CAF (*p* < 0.001) and CAF + E (*p* = 0.004). In contrast, for women, only CAF + CE had a higher BHI compared to CAF (*p* = 0.003) and CAF + E (*p* = 0.037), whereas water was not different from either CAF or CAF + E when averaged across time points. At 240 min, there was both a drink (*p* < 0.001) and drink x sex interaction (*p* = 0.042), with women exhibiting higher BHI than men (*p* = 0.03) for caffeinated drinks despite dosages of higher relative fluid and caffeine. As shown in Figure 3B, at the end of the protocol, CAF but not CAF + CE was considered inferior to the water BHI standard of 1.0. Despite inter-individual variability, the mean difference for CAF from 1.0 for men (−0.20) was significant (*p* < 0.001) and 95%CI lower bounds [−0.33, −0.083] well below clinical relevance, with a large effect size (d = −1.3). Women also showed CAF to be inferior to the BHI of 1.0 (*p* = 0.049, 95%CI [−0.16, −0.001), with a moderate effect size (d = −0.56). When considering CAF + CE at the end of the protocol, the difference from 1.0 was non-significantly higher for women (*p* = 0.09, 95% CI [−0.01, 0.12] with a small to moderate benefit (d = 0.48) and non-significantly (*p* = 0.18, 95% CI [−0.11, 0.02]) lower than 1.0 for men with a small negative effect (d = −0.38).

Habitual caffeine intake effects on BHI. There was no drink × habitual use interaction effect (*p* = 0.81). Habitual intake status had no effect on the BHI (*p* = 0.827) between the naïve (0.92 ± 0.1) and habitual user group (0.93 ± 0.1) averaged across all three caffeinated beverages over 120 and 240 min. After 240 min, mean (±SD) BHIs for CAF, CAF + E, and CAF + CE were 0.86 ± 0.17, 0.89 ± 0.14, and 0.99 ± 0.06 for naïve (*n* = 19) and 0.86 ± 0.16, 0.91 ± 0.17, and 1.02 ± 0.14 for habitual users (*n* = 9), respectively.

### 3.2. Net Fluid Balance, Urine Mass and Fluid Retention

**Figure 4:**
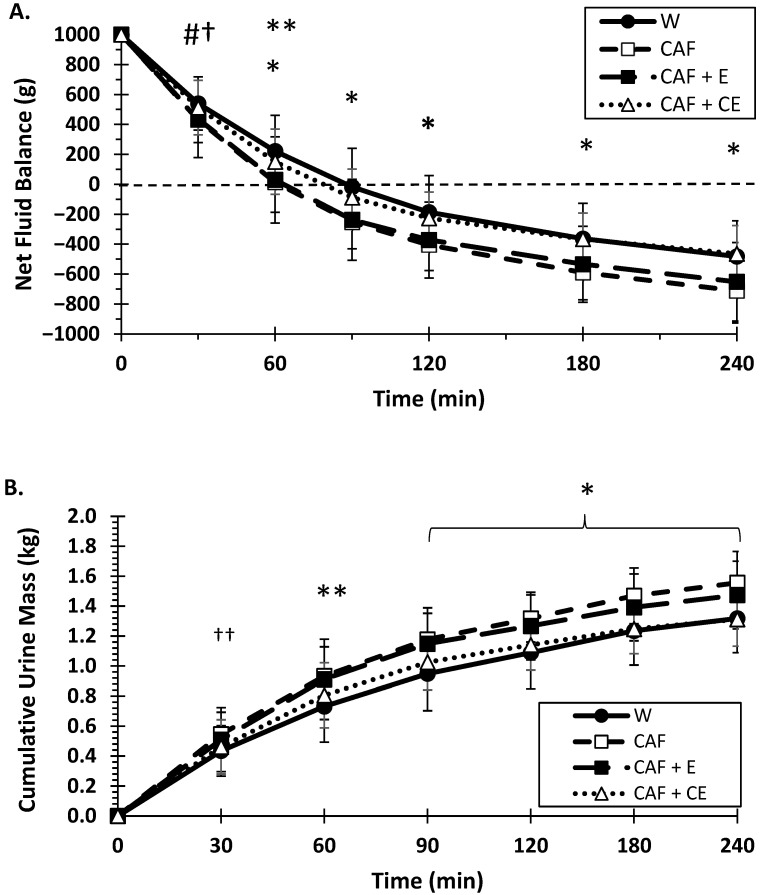
Mean (±SD) net fluid balance (**A**) and cumulative urine mass (**B**) during 240 min for W (water); CAF (caffeine); CAF + E (caffeine + electrolytes); and CAF + CE (caffeine + carbohydrate+ electrolytes) # Water > CAF and CAF + E (*p* < 0.05) at 30 min (more positive net fluid balance). † CAF + CE > CAF (*p* = 0.05) and †† Both Water and CAF + CE > CAF at 30 min. * Water and CAF + CE different than CAF and CAF + E (*p* < 0.05). ** Water > all beverages (*p* < 0.05).

As presented in Figure 4A, net fluid balance (NFB) had significant drink effects (*p* < 0.001) and drink × time interaction (*p* < 0.001). Water and CAF + CE averaged a more positive fluid balance (*p* < 0.001, *p* < 0.001) compared to CAF and CAF + CE, ranging from a 120 to 175 mL greater overall fluid balance. Beginning at 60 min, water had a more positive net fluid balance than all other energy beverages, while CAF + CE also exerted a more positive NFB compared to CAF and CAF + E from 90 until 240 min. After 90 min, all beverages elicited a negative NFB: water (−17.6 mL ± 258.8), CAF (−253.5 mL ± 177.2), CAF + E (−236.6 mL ± 270.8), and CAF + CE (−88.5 mL ± 191.3). Except at 60 min, there was no difference between water and CAF + CE; moreover, at no point in time was there a difference between CAF and CAF + E in NFB.

Figure 4B illustrates changes in cumulative urine mass over time. There were significant drink (*p* = 0.000) and drink × time interaction (*p* = 0.000) effects for urine mass accumulated (Figure 4B). Overall, water and CAF + CE averaged less urine output (*p* < 0.001, *p* = 0.001) versus CAF and CAF + E. There was no significant difference (*p* = 0.68) in urine output between water vs. CAF + CE and no difference between CAF vs. CAF + E (*p* = 0.69). From 60 until 240 min, water and CAF + CE had lower urine output compared to CAF (*p* < 0.05) and CAF + E (*p* < 0.02), except at 60 min, where water had lower diuresis compared to CAF + CE (by 74 g) (*p* = 0.042) along with CAF (by 201 g) and CAF + E (by 180 g). At 4 h, compared to CAF + CE, urine mass was 244 g greater (*p* < 0.001) for CAF and 162 g greater (*p* < 0.001) for CAF + E. Note that a mean difference of 244 g less urine output is equivalent to ~25% of the original 1000 mL of fluid given.

**Figure 5:**
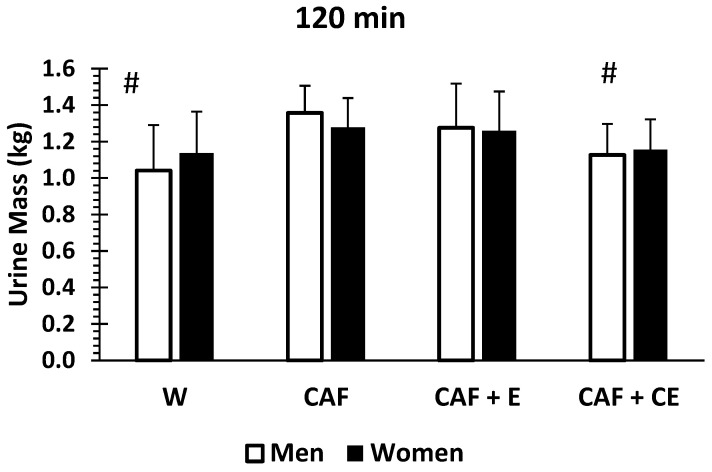

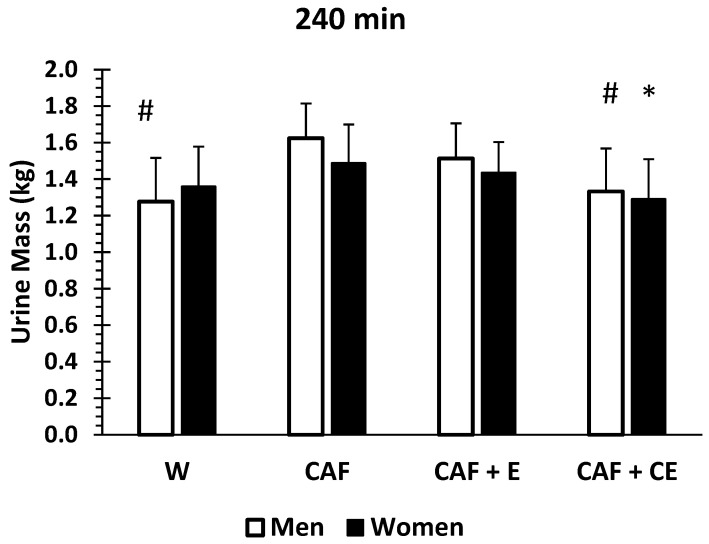
Mean (±SD) urine mass at 120 (**top**) and 240 min (**bottom**) for men and women based on beverages W (water); CAF (caffeine); CAF + E (caffeine + electrolytes); and CAF + CE (caffeine + carbohydrates + electrolytes. # Water and CAF + CE < CAF and CAF + CE (in men at 2, 4 h) (*p* < 0.05). * CAF + CE < CAF and CAF + CE (in women only at 240 min) (*p* < 0.05).

There was no sex difference for the impact of drinks on urine mass diuresis (*p* = 0.77), or sex x drink interaction (*p* = 0.051). As presented in Figure 5, by 240 min, men and women (averaged across all drinks) only differed by 52 g (1.437 kg ± 0.21 and 1.385 kg ± 0.20 of urine mass loss, respectively). However, men and women had different diuretic responses to the energy drinks compared to water. For men at 120 and 240 min, water and CAF + CE had significantly less urine output than CAF (*p* < 0.001) and CAF + E (*p* < 0.001). In contrast, for women, there were no differences in urine output among the four drinks at 120 min; however, at 240 min, only CAF + CE was less than CAF (*p* = 0.007) and CAF + E (*p* = 0.015) by 196 g and 144 g, respectively. Additionally, water was not different compared to any other energy drink in women at 240 min.

The % fluid retained over time is presented below in Table 4. There was a significant drink (*p* < 0.001) and drink × time interaction (*p* < 0.001) for % fluid retained. Overall, water and CAF + CE had a greater % fluid retained than CAF and CAF + E (*p* < 0.001). At 30 min, water was better retained than CAF (*p* = 0.008) and CAF + E (*p* = 0.038), but CAF + CE was only better retained compared to CAF (*p* = 0.048). One hour after ingestion, all beverages were still positive, but not similar in the % of ingested fluid that was retained. Water continued to have greater retention compared to CAF and CAF + E after 60 min and also greater than CAF + CE (*p* = 0.023), but only at 60 min. After 90 min and until 240 min, water and CAF + CE had a greater % fluid retained than CAF and CAF + E (*p* < 0.005).

### 3.3. Urine Osmolality

**Figure 6:**
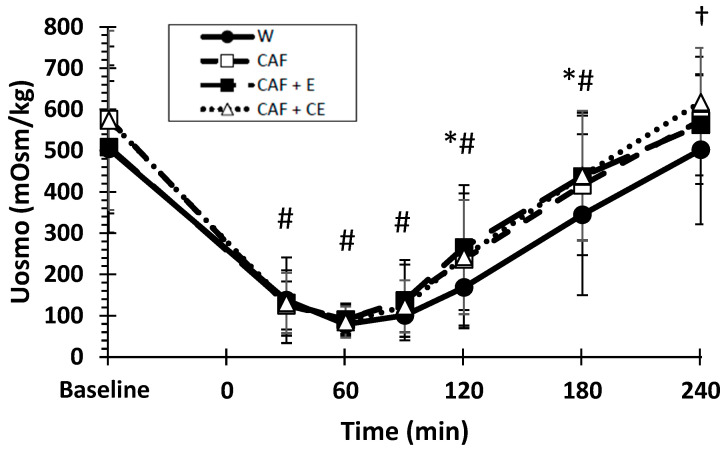
Mean (±SD) urine osmolality (UOsm) over time, denoting significant differences between beverages. * Water < CAF + CE and CAF + E (*p* < 0.05). † Water < CAF + CE (*p* = 0.005). # Baseline > 30, 60, 90, 120, and 180 min (*p* < 0.02).

There was a significant drink effect (*p* < 0.001) and drink × time interaction (*p* = 0.010) for urine osmolality (Figure 6). On average, water was significantly lower than CAF + CE (*p* = 0.003) and CAF + E (*p* = 0.028). Water was lower than CAF + CE and CAF + E at 2 h (*p* = 0.005, 0.001, respectively) and 3 h (*p* = 0.010, 0.038, respectively). At 4 h, water was only lower than CAF + CE (*p* = 0.005). There was also a significant time effect compared to baseline with lower osmolality at 30, 60, 90, 120, and 180 min (*p* < 0.02).

### 3.4. Thirst Rating

Based on a seven-point Likert scale, there was no drink effect (*p* = 0.057) or drink × time interaction (*p* = 0.33). There was a time effect (*p* < 0.001) with lower thirst immediately after drinking and 60 min post-ingestion. Thirst was similar at baseline to the post-ingestion rating at 2 and 3 h. Thirst was greater than baseline at 4 h (*p* = 0.042).

## 4. Discussion

The present study compared caffeinated energy drink formulations using a modified beverage hydration index approach (equal volume of water ingested along with the energy drink). This approach provided ecological validity as the volume of energy drink ingested was analogous to a large serving (~1 can) of several commercially available energy drink products. Notably, the CAF + CE beverage, containing a moderate caffeine dose (~106 mg), carbohydrates, and a higher sodium concentration (~44.7 mmol), demonstrated hydration properties statistically indistinguishable from water across multiple measures, including BHI, net fluid balance, and cumulative urine output. This finding is particularly relevant given the formulation’s similarity to oral rehydration solutions (ORS), which are designed to enhance intestinal water absorption and fluid balance. The CAF + CE did not compromise fluid balance when compared to water, but it also did not improve it, as would be expected with a true ORS. Thus, these results support the potential utility of such formulations for individuals seeking an alternative form of hydration similar to water without compromising fluid balance, especially in contexts where caffeine intake is desired or unavoidable. However, in contrast, energy drinks consisting of 280 mg caffeine with limited glucose and/or electrolytes had lower hydration properties compared to water. Furthermore, we observed that, in contrast to our stated hypothesis, women appeared to retain more of caffeinated energy drinks (i.e., less urine output and higher BHI) in comparison to men. This unexpected sex difference was observed despite women consuming higher relative fluid and caffeine dosages versus men (when adjusted for body size). Moreover, in contrast to prevailing thought and our own stated hypothesis, habitual caffeine status (and presumably caffeine tolerance) had no significant impact on caffeine energy drink diuretic actions in a resting experimental model utilizing the BHI.

Our findings regarding the impact of caffeine on BHI under resting conditions (i.e., non-exercise) are, for the most part, consistent with much of the literature. The BHI for caffeinated beverages evaluated in the original investigation [10] were tea, coffee, and diet cola. Although these beverages returned a BHI < 1.0 (indicating values suggestive of less hydration relative to water), this difference was not statistically significant [10]. Sugar-sweetened cola (with a similar caffeine content as CAF + CE but with higher sugar and negligible sodium) had a BHI slightly >1.0, although again, this beverage was not significantly higher than water. Only milks, orange juice, and commercially available beverages considered to be ORS were significantly higher than water [10], along with subsequent studies yielding BHI values averaging >1.2–1.5 [16,31]. The addition of carbohydrates and electrolytes to beverages also tends to produce a higher BHI compared to electrolytes alone [29]. ORS beverages contain macronutrients (~2% carbohydrate) along with higher electrolyte levels (e.g., sodium > 30 up to 55 mmol) than a sports drink (~20 mmol sodium). The WHO ORS formula recommends an even higher range with 75 mmol of Na^+^ to equate to glucose levels (75 mmol).

Therefore, the question we probed was whether caffeinated beverages claiming to represent the hydration properties of ORS can be similar to or better than water. And does the presence of caffeine obscure the potential benefits of an ORS-type formula? Using a systematic approach, Maughan and colleagues [11] reported that increasing the carbohydrate content above 5% (to 10 and 20%) improved fluid retention of beverages using the BHI protocol; however, increasing caffeine from 100 to 400 mg had no effect. Therefore, our results did not concur with significantly lower BHI (<1.0 water standard) in CAF and CAF + E despite these beverages being in the caffeine range (280 mg) of that previous study. The fact that we diluted the impact of the energy drinks (500 mL) with equal volumes of water (500 mL) may explain the differential findings but may be more representative of real-world beverage consumption (i.e., 1 L of energy drinks not typically consumed in a single sitting). Therefore, whether the improved hydration with CAF + CE over CAF and CAF + E can be attributed to lower caffeine content combined with the inclusion of carbohydrates and electrolytes (more analogous to ORS) is unknown. Unfortunately, a direct comparison of the CAF + CE formula without caffeine was not available to us.

In contrast to previous reports [15,16], women had a higher overall average BHI (across all four beverages) with a 7.7% higher ratio compared to men, suggesting better fluid retention. This occurred despite the fact that women consumed greater fluid and caffeine dosages relative to body mass. Increased estrogen and progesterone (either via oral contraceptives or during the luteal phase in normal menstruation) have independent effects on hydration, typically leading to increased fluid retention [32,33]. The effect of female hormones on the hydration potential of caffeinated beverages has not been directly examined. One study [34] suggested that women on oral contraceptives may have reduced caffeine clearance (prolonged blood caffeine half-life) compared to women not on oral contraceptives. Caffeine elimination may also be slower in the late luteal phase, leading to greater accumulation, although the authors acknowledged [35] that the effect may be too small to be of clinical significance in most women. We did not inventory our female participants regarding menstrual cycle phase/status, and only three of fourteen listed oral contraceptive use. Whether sex-based differences currently observed are linked to hormonal fluctuations is unclear [36] or alternatively attributed to individual variability in the rate-limiting enzyme in caffeine metabolism. Fluid balance and hydration considerations for women remain to be examined further and are likely better addressed with improved control in hormonal status for women participating in future investigations.

Habituation to caffeine intake (tolerance) has previously been identified to attenuate the diuretic actions of caffeine under resting conditions [12,14]. The threshold needed for establishing this tolerance is unclear, particularly when relying upon survey methods that are not necessarily validated for the population [17]. Our “habitual user” group also tended to include a majority of participants whose typical caffeine intake would be categorized as “mild” [17], with an average of ~156 mg per day or <3 g/kg. However, this level of habitual caffeine intake appears representative compared to reports on college students of ~173 mg/day [37]. Since the range of caffeine intakes was limited (and low in our subject pool), a correlation between habitual intake and urine mass from CAF was R^2^ = 0.023, *p* = 0.43, an essentially flat line of association. Nonetheless, future studies might better address the impact of habitual caffeine use on diuresis using the BHI test protocol with a greater range of habitual users, especially including those on the “higher” end of habitual caffeine use (6–9 mg/kg per day).

### Limitations

To evaluate the impact of caffeine habituation status, we had an unbalanced study design with unequal cell sizes for naïve and habituated groups; moreover, the range of daily caffeine intake was concentrated toward a low or below-average intake (<250 mg). This somewhat limits the generalizability of our results, although it refutes the previous premise that caffeine-naïve individuals exhibit greater diuresis with caffeine at rest. The use of commercially available products did not permit the control of the caffeine levels and other components across all three beverages; thus, the study design was unable to systematically evaluate the interaction between the level of caffeine and electrolytes/sugar across test beverages since the caffeine level was not held constant. Whether the dose of caffeine or the addition of sugar and/or electrolytes is responsible for improved beverage hydration potential cannot be ascertained. Likewise, additional substances (e.g., taurine, glucuronolactone, theanine, vitamins) may or may not be present in energy drink products and, if present, have unknown renal handling effects at the dosages contained.

The BHI model, by nature, also does not allow for the evaluation of intestinal absorption rates of beverages but utilizes fluid retention as a renal marker of hydration potential. Whether decreased urine output (and a higher BHI index) is influenced more by greater water retention or a slower entry of water across the gastrointestinal tract (particularly within the first hour following ingestion) cannot be ascertained. Furthermore, another limitation was that no other meaningful impacts (along with beverage hydration properties) for potential outcomes on behavioral, cognitive, or physiological (e.g., blood pressure) or physical performance measures were examined. Future studies should investigate these additional impacts during BHI studies.

Finally, a modification of the beverage hydration index protocol was used (500 mL of water of the total 1 L substituted for a 500 mL test beverage) due to concerns regarding the caffeine level and ecological validity (ingesting 1 L of an energy drink in 30 min). This limits the ability to place energy drinks as a “beverage category” on the original continuum established by Maughan et al. [10] to compare with previous studies that included other caffeinated beverage types (e.g., teas, coffees, and colas). The reality, however, is that even if the full one liter of test beverage had been used, the variability of caffeine in energy drink products (reported from 33 to 140 mg) [21] and other substances preclude a generalized BHI rating.

## 5. Conclusions

A low-carbohydrate energy beverage with ~100 mg caffeine and electrolytes (analogous to an ORS) had similar hydration properties compared to water; however, in contrast, caffeinated drinks of 280 mg with limited glucose and/or electrolytes had lower hydration properties (≥−14%) compared to water. Contrary to our hypothesis, an individual’s habitual caffeine status had no impact on diuresis when consuming up to 280 mg caffeine. Moreover, a novel finding was that women experienced greater retention of caffeinated energy drinks compared to men despite consuming greater relative amounts of both fluid and caffeine (based on their smaller body size). Practical applications from these results suggest that not all energy drinks containing varying amounts of caffeine, electrolytes, and other ingredients are equivalent in their hydration properties and diuretic effects. However, individuals who are caffeine-naïve do not appear to experience a greater adverse impact on fluid balance after consuming caffeinated energy drinks along with additional water.

## Figures and Tables

**Table 1:** Participant mean (±SD) characteristics. * indicates sex difference (*p* < 0.05).

	Men (*n* = 14)	Women (*n* = 14)	Total (*N* = 28)
Age (yr)	23.9 ± 3.7	21.3 ± 2.4	22.6 ± 3.3
Height (cm)	181.1 ± 7.6 *	164.6 ± 6.7	172.8 ± 10.9
Mass (kg)	75.8 ± 9.2 *	61.0 ± 7.4	68.4 ± 11.1
% Body Fat—Skinfold	11.4 ± 5.0 *	21.2 ± 4.3	16.3 ± 6.7
Body Mass Index	23.1 ± 2.3	22.5 ± 2.1	22.8 ± 2.2
Fat Free Mass (kg)—Skinfold	67.1 ± 7.9 *	48.0 ± 5.6	57.6 ± 11.8
Total Body Water (kg)—Skinfold	50.5 ± 5.9 *	36.9 ± 4.2	43.7 ± 8.6
Total Body Water (kg)—InBody	47.5 ± 6.3 *	32.9 ± 5.0	40.2 ± 9.3

**Table 2:** Beverage composition as measured.

Beverage	Osmolality (mOsm)	Sodium (mmol)	Potassium (mmol)	Carbohydrate (gm/500 mL)	Caffeine (mg/500 mL)
Water	2.5 ± 0.8	0.5 ± 0.0	0.1 ± 0.0	0	0
CAF	98.3 ± 3.6	0.6 ± 0.1	0.6 ± 0.1	0	280
CAF + E	139.9 ± 4.4	8.1 ± 0.8	10.4 ± 1.0	4	280
CAF + CE	225.3 ± 3.8	44.7 ± 2.5	18.0 ± 1.2	12	106

**Table 3:** Mean (±SD) dietary intake 24 h prior to each experimental trial (*N* = 28). Water intake is based on food and beverages. Nutrient intakes were not different (*p* > 0.05) across treatments.

Nutrient	WAT	CAF	CAF + E	CAF + CE
Water (g)	4103 ± 1585	4088 ± 1682	4140 ± 1592	4316 ± 1701
Energy (kcal)	2324 ± 899	2428 ± 724	2384 ± 926	2249 ± 686
Sodium (mg)	2617 ± 1375	3475 ± 1693	3144 ± 1694	3040 ± 1808
Protein (g)	120 ± 67	116 ± 46	127 ± 81	119 ± 58
Carbohydrate (g)	284 ± 108	276 ± 82	287 ± 116	264 ± 80

**Table 4:** Mean ± SD % fluid retained (of original 1 L ingested) over the beverage hydration index protocol. * Significantly greater % fluid retention vs. CAF & CAF +E. † Significantly greater than all drinks (CAF, CAF + E, and CAF + CE).

Time (min)	Water	CAF + CE	CAF	CAF + E
60	22.2 ± 3.9 †	15.1 ± 21.8 *	1.4 ± 20.0	3.0 ± 28.9
90	−1.8 ±25.9 *	−8.9 ± 19.1 *	−25.4 ± 17.7	−23.7 ± 27.1
120	−18.7 ± 24.5 *	−22.6± 17.5 *	−40.5 ± 17.1	−37.2 ± 25.4
180	−36.3 ± 3.7 *	−36.6± 17.3 *	−59.0 ± 18.2	−53.4 ± 25.4
240	−48.2 ±3.8 *	−46.4 ±18.8 *	−71.2 ± 21.5	−65.1 ± 26.3

## Data Availability

The original contributions presented in this study are included in the article. Further inquiries can be directed to the corresponding author.

## References

[1] Institute of Medicine (2005). Dietary Reference Intakes for Water, Potassium, Sodium, Chloride, and Sulfate.

[2] Nissensohn M., Castro-Quezada I., Serra-Majem L. (2013). Beverage and water intake of healthy adults in some European countries. Int. J. Food Sci. Nutr..

[3] Maughan R., Owen J., Shirreffs S., Leiper J. (1994). Post-exercise rehydration in man: Effects of electrolyte addition to ingested fluids. Eur. J. Appl. Physiol. Occup. Physiol..

[4] Shirreffs S.M., Taylor A.J., Leiper J.B., Maughan R.J. (1996). Post-exercise rehydration in man: Effects of volume consumed and drink sodium content. Med. Sci. Sports Exerc..

[5] Ray M.L., Bryan M.W., Ruden T.M., Baier S.M., Sharp R.L., King D.S. (1998). Effect of sodium in a rehydration beverage when consumed as a fluid or meal. J. Appl. Physiol..

[6] Evans G.H., James L.J., Shirreffs S.M., Maughan R.J. (2017). Optimizing the restoration and maintenance of fluid balance after exercise-induced dehydration. J. Appl. Physiol..

[7] Osterberg K.L., Pallardy S.E., Johnson R.J., Horswill C.A. (2010). Carbohydrate exerts a mild influence on fluid retention following exercise-induced dehydration. J. Appl. Physiol..

[8] Maughan R.J., Leiper J.B., Shirreffs S.M. (1996). Restoration of fluid balance after exercise-induced dehydration: Effects of food and fluid intake. Eur. J. Appl. Physiol. Occup. Physiol..

[9] Mitchell D.C., Knight C.A., Hockenberry J., Teplansky R., Hartman T.J. (2014). Beverage caffeine intakes in the U.S.. Food Chem. Toxicol..

[10] Maughan R.J., Watson P., Cordery P.A., Walsh N.P., Oliver S.J., Dolci A., Rodriguez-Sanchez N., Galloway S.D. (2016). A randomized trial to assess the potential of different beverages to affect hydration status: Development of a beverage hydration index. Am. J. Clin. Nutr..

[11] Maughan R.J., Watson P., Cordery P.A.A., Walsh N.P., Oliver S.J., Dolci A., Rodriguez-Sanchez N., Galloway S.D.R. (2019). Sucrose and Sodium but not Caffeine Content Influence the Retention of Beverages in Humans Under Euhydrated Conditions. Int. J. Sport Nutr. Exerc. Metab..

[12] Maughan R.J., Griffin J. (2003). Caffeine ingestion and fluid balance: A review. J. Hum. Nutr. Diet..

[13] Marx B., Scuvée É., Scuvée-Moreau J., Seutin V., Jouret F. (2016). Mechanisms of caffeine-induced diuresis. Med. Sci..

[14] Armstrong L.E. (2002). Caffeine, body fluid-electrolyte balance, and exercise performance. Int. J. Sport Nutr. Exerc. Metab..

[15] Zhang Y., Coca A., Casa D.J., Antonio J., Green J.M., Bishop P.A. (2015). Caffeine and diuresis during rest and exercise: A meta-analysis. J. Sci. Med. Sport.

[16] Sollanek K.J., Tsurumoto M., Vidyasagar S., Kenefick R.W., Cheuvront S.N. (2018). Neither body mass nor sex influences beverage hydration index outcomes during randomized trial when comparing 3 commercial beverages. Am. J. Clin. Nutr..

[17] Filip A., Wilk M., Krzysztofik M., Del Coso J. (2020). Inconsistency in the Ergogenic Effect of Caffeine in Athletes Who Regularly Consume Caffeine: Is It Due to the Disparity in the Criteria That Defines Habitual Caffeine Intake?. Nutrients.

[18] Penna E.M., Harp A., Hack B., Talik T.N., Millard-Stafford M. (2024). Guarana (*Paullinia cupana*) but Not Low-Dose Caffeine Improves Cycling Time-Trial Performance Versus Placebo. Int. J. Sport Nutr. Exerc. Metab..

[19] Watson E.J., Kohler M., Banks S., Coates A.M. (2017). Validation and reproducibility of an Australian caffeine food frequency questionnaire. Int. J. Food Sci. Nutr..

[20] McCusker R.R., Goldberger B.A., Cone E.J. (2003). Caffeine content of specialty coffees. J. Anal. Toxicol..

[21] McCusker R.R., Goldberger B.A., Cone E.J. (2006). Caffeine content of energy drinks, carbonated sodas, and other beverages. J. Anal. Toxicol..

[22] Wikoff D., Welsh B.T., Henderson R., Brorby G.P., Britt J., Myers E., Goldberger J., Lieberman H.R., O’Brien C., Peck J. (2017). Systematic review of the potential adverse effects of caffeine consumption in healthy adults, pregnant women, adolescents, and children. Food Chem. Toxicol..

[23] Jackson A.S., Pollock M.L. (1978). Generalized equations for predicting body density of men. Br. J. Nutr..

[24] Jackson A.S., Pollock M.L., Ward A. (1980). Generalized equations for predicting body density of women. Med. Sci. Sports Exerc..

[25] Wang Z., Deurenberg P., Wang W., Pietrobelli A., Baumgartner R.N., Heymsfield S.B. (1999). Hydration of fat-free body mass: Review and critique of a classic body-composition constant. Am. J. Clin. Nutr..

[26] World Health Organization (2002). Reduced Osmolarity: Oral Rehydration Salts (ORS) Formulation: A Report from a Meeting of Experts Jointly Organised by UNICEF and WHO.

[27] Atia A.N., Buchman A.L. (2009). Oral rehydration solutions in non-cholera diarrhea: A review. Am. J. Gastroenterol..

[28] Pence J., Bloomer R.J. (2020). Impact of Nuun Electrolyte Tablets on Fluid Balance in Active Men and Women. Nutrients.

[29] Millard-Stafford M., Snow T.K., Jones M.L., Suh H. (2021). The Beverage Hydration Index: Influence of Electrolytes, Carbohydrate and Protein. Nutrients.

[30] Tinsley G.M., Siedler M.R., Rodriguez C., Harty P.S., Stratton M.T., White S.J., Keith D.S., Green J.J., Boykin J.R., Williams A.D. (2023). Evaluation of novel beverage formulations for hydration enhancement in humans. J. Electr. Bioimpedance.

[31] Clarke M.M., Stanhewicz A.E., Wolf S.T., Cheuvront S.N., Kenefick R.W., Kenney W.L. (2019). A randomized trial to assess beverage hydration index in healthy older adults. Am. J. Clin. Nutr..

[32] Stachenfeld N.S. (2008). Sex hormone effects on body fluid regulation. Exerc. Sport Sci. Rev..

[33] Stachenfeld N.S., Silva C., Keefe D.L., Kokoszka C.A., Nadel E.R. (1999). Effects of oral contraceptives on body fluid regulation. J. Appl. Physiol..

[34] Abernethy D.R., Todd E.L. (1985). Impairment of caffeine clearance by chronic use of low-dose oestrogen-containing oral contraceptives. Eur. J. Clin. Pharmacol..

[35] Lane J.D., Steege J.F., Rupp S.L., Kuhn C.M. (1992). Menstrual cycle effects on caffeine elimination in the human female. Eur. J. Clin. Pharmacol..

[36] Rodriguez-Giustiniani P., Rodriguez-Sanchez N., Galloway S.D.R. (2022). Fluid and electrolyte balance considerations for female athletes. Eur. J. Sport Sci..

[37] Mahoney C.R., Giles G.E., Marriott B.P., Judelson D.A., Glickman E.L., Geiselman P.J., Lieberman H.R. (2019). Intake of caffeine from all sources and reasons for use by college students. Clin. Nutr..

